# Computational Modeling of the Interplay between Cadherin-Mediated Cell Adhesion and Wnt Signaling Pathway

**DOI:** 10.1371/journal.pone.0100702

**Published:** 2014-06-26

**Authors:** Jiawen Chen, Zhong-Ru Xie, Yinghao Wu

**Affiliations:** Department of Systems and Computational Biology, Albert Einstein College of Medicine of Yeshiva University, Bronx, New York, United States of America; Vanderbilt University, United States of America

## Abstract

Wnt signaling and cadherin-mediated adhesion have been implicated in both processes of embryonic development and the progression of carcinomas. Recent experimental studies revealed that Wnt signaling and cadherin-mediated cell adhesion have close crosstalk with each other. A comprehensive model that investigates the dynamic balance of β-catenins in Wnt signaling and cell adhesion will improve our understanding to embryonic development and carcinomas. We constructed a network model to evaluate the dynamic interplay between adhesion and Wnt signaling. The network is decomposed into three interdependent modules: the cell adhesion, the degradation circle and the transcriptional regulation. In the cell adhesion module, we consider the effect of cadherin’s lateral clustering. We found adhesion negatively contributes to Wnt signaling through competition for cytoplasmic β-catenins. In the network of degradation circle, we incorporated features from various existing models. Our simulations reproduced the most recent experimental phenomena with semi-quantitative accuracy. Finally, in the transcriptional regulation module, we developed a function selection strategy to analyze the outcomes of genetic feedback loops in modulating the gene expression of Wnt targets. The specific cellular phenomena such as cadherin switch and Axin oscillation were archived and their biological insights were discussed. Our model provides the theoretical basis of how spatial organization regulates the dynamics of cellular signaling pathways. We suggest that cell adhesion affects Wnt signaling in both negative and positive ways. Cadherins can inhibit Wnt signaling not only in a way as a stoichiometric binding partner of β-catenins that sequesters them from signaling, but also in a way through their clustering to impacts the rate at which β-catenins are involved in the destruction loop. Additionally, cadherin clustering increases the phosphorylation rate of β-catenins and promotes its signaling in nucleus.

## Introduction

The Wnt signaling pathway has crucial roles in the normal processes of embryonic development [1], [2]. The mutations or deregulated expressions of its components lead to multiple diseases such as cancer metastasis [3]–[6]. In both embryonic development and cancer metastasis, the extracellular Wnt signals can activate the initially immobile cells to lose their polarized traits and acquire highly motile characteristics, generally known as epithelial-mesenchymal transition (EMT) [7]–[9]. One of the most remarkable hallmarks of EMT is the repression of cadherin-mediated cell adhesion [10], [11]. Recent experimental studies have demonstrated that Wnt signaling and cadherin-mediated cell adhesion interplay with each other through multiple pathways [12]–[15]. A comprehensive model that investigates the interplay between these two processes will provide useful information to understand EMT and cancer metastasis.

The gene expression of the Wnt signaling targets is regulated by the cytoplasmic distributions of β-catenins, a central component in the Wnt signaling network [16]–[18]. In the absence of Wnt ligands, β-catenins are phosphorylated in the destruction complexes consisting of axins and APCs, which results in the degradation of β-catenins in proteasome [19]. In the presence of Wnt ligands, on the other hand, Axins are recruited to the plasma membrane, leading to the dysfunction of destruction complexes. The inhibition of degradation stabilizes β-catenins, gives them higher probability to translocate into the cell nucleus, where they can activate the Wnt target genes as a co-transcriptional factor. Not only can β-catenins switch the Wnt signaling pathway between “on” and “off” states, but they can also bind to the cytoplasmic domain of classic cadherins as part of the cell adhesive complex [20], [21]. The dynamic balance of β-catenin in degration, signaling and adhesion is closely regulated by phosphorylation [22]. The balance plays critical roles in making decision of cellular fate, although the detailed mechanism is not well understood.

Recent progresses in experimental studies of Wnt signaling pathway begin to throw light on how the cytoplasmic pool of β-catenins is maintained and regulated, and how the destructive, signaling or adhesive pathway of β-catenin can interplay with each other. The study on endogenous destruction complex indicated that level of phosphorylated beta-catenin remains unchanged in ‘Wnt on’ scene [23]. Detailed kinetic studies demonstrated that level of phosphorylated beta-catenin decreases in 0.5 hr after Wnt treatment, then it recovers to its initial level after 2 hr [24]. Kim and co-authors further showed that the ability of LRP6 binding to Axin increase in the first 0.5 hr and decrease to its initial level after 2 hr [25]. All of these new experimental observations have not been systematically assessed by current theoretical models. An updated Wnt signaling model that could explain these new experimental phenomena is highly demanded. On the other side, there are interesting new experimental results that directly investigate the interplay between Wnt signaling and cadherin-mediated cell adhesion. In additional to our recent studies on the mechanism of cadherin clustering during cell adhesion, Maher et al discovered that the kinetics of β-catenin degradation in the membrane-associated destruction complex is enhanced by cadherin-based cell adhesion [14]. Moreover, Howard et al have demonstrated that mutating β-catenin at residue Y654 decreases expression of Wnt target gene, which indicates that cadherin-mediated adhesion positively contributes to Wnt signaling [15]. Finally, APC and Axin which participate in the destruction complex have also been detected to retain β-catenins in the cytoplasmic region [26].

Comparing with experimental studies, computational modeling and theoretical analysis possess of unique advantages. They allow one to test conditions that may currently be difficult to attain or even unapproachable in the laboratory. During the past decade, a variety of mathematical or computational models have been applied to study the Wnt signaling pathway at different levels [27]. The first quantitative model of the canonical Wnt pathway was proposed by Lee et al by a system of coupled ODEs to describe how the key proteins in the pathway change over time in response to the Wnt stimulation [28]. This model was later tested and validated under different circumstances [29]–[34]. Further extension has been applied to analyze the effect of mutations [35] and the crosstalk between Wnt and other signaling pathways [36]. Since most of these applications are based on Lee’s model, in which activities of destruction complexes were inhibited under Wnt stimulation, they are difficult to explain some of the very recent experimental results. Besides that, the importance of phosphorylation in regulation of cytoplasmic pool of β-catenins was not fully considered. Additionally, subcellular models were developed to study the intracellular localization of protein complex in the pathway, for instance, the nucleo-cytoplasmic shuttling of APC [37]. Efforts have also been made to link Wnt signaling to the changes of inter-cellular interactions. They focused on the relation between the function of β-catenin and cadherin-based cell adhesion [38], although the detailed process of cadherin clustering was not taken into accounts. At the multicellular level, hybrid models were developed to understand the functional roles of Wnt signaling pathway in EMT [39], cell proliferation [40] and adhesion in intestinal crypts [41], and cell fate determination [42].

Here we constructed a network model to reconsider the dynamics of Wnt signaling pathway by integrating the most updated experimental information. The network is decomposed into three interdependent modules: the cell adhesion, the degradation circle and the transcriptional regulation. In the degradation circle module, we applied the “Axin inactivation” model to describe the degradation pathway of β-catenins and the interactions between destruction complexes and Wnt receptors. In the cell adhesion module, we also consider the effect of cadherin’s lateral clustering, which leads to the formation of cellular junctions and modulates their cytoplasmic interactions with β-catenins. In the transcriptional regulation module, we developed a function selection strategy to analyze the outcomes of genetic feedback loops in regulating the gene expression of Wnt targets such as cadherin and Axin. In these modules, the spatial information of protein interactions and the effects of phosphorylation are taken into account. These new features allow us to investigate the interplay between cell adhesion and Wnt signaling in both molecule scale (like chemical reactions) and sub-cellular scale (like transcription feedback). We explored if cadherin clustering play other regulatory roles, in addition to simply compete β-catenins with Wnt signaling pathway. The effects of mutations in network components have also been estimated. Overall, this work will help us form a comprehensive understanding towards the molecular mechanism of Wnt signaling pathway.

## Models

### Overall description of the model framework

**Figure 1:**
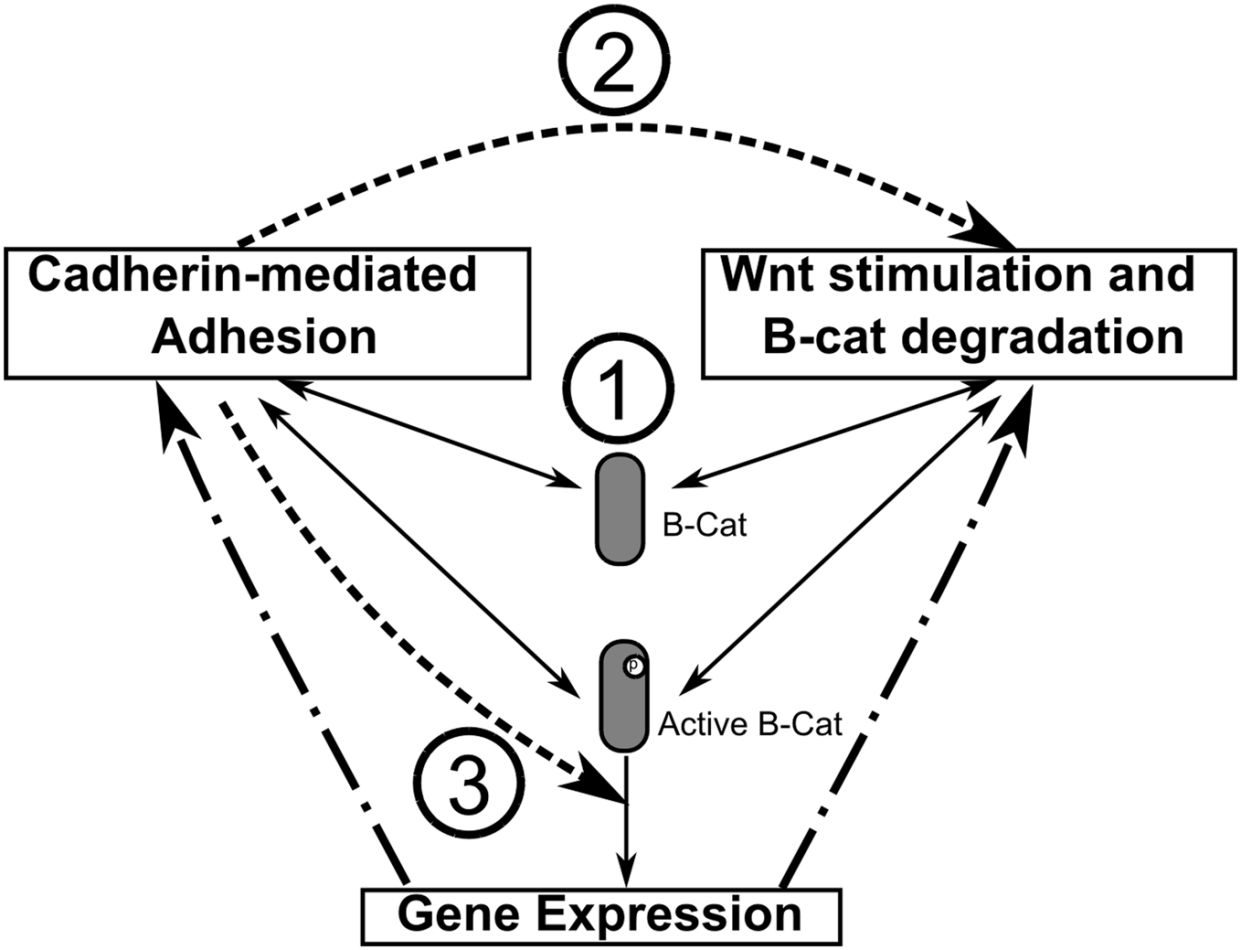
The overall representation of our network model. There are three modules in the network. These modules are connected by two distinctive forms of β-catenin. In additional to the normal form of β-catenins, a second form, active β-catenin, is presented in our model. Only the active β-catenins can activate the gene expression. The activated genetic pathways further affect expressions of proteins in both cadherin adhesion and β-catenin degradation modules through genetic feedback loops. The interplay between Wnt signaling and cell adhesion is captured by inter-module crosstalk, which are labeled with numbers.

There are three modules in the network (**Fig. 1**). The cell adhesion module describes the dynamics of adherens junction formation and the interactions between cadherins and β-catenins during this process. The Wnt activation and β-catenin degradation module gives the connection between extracellular activation of Wnt ligands and intracellular loop of β-catenin degradation. The transcriptional regulations of Wnt target genes are enclosed in the Wnt target gene expressions module. These three modules are connected by β-catenins. The dynamics of the entire network therefore is regulated by the balance of β-catenins in different modules. As shown in the figure, there are two distinctive forms of β-catenin pools shared between adhesion and degradation modules. In additional to the normal form of β-catenins, a second form, active β-catenin, is presented in our model. Only the active β-catenins can activate the gene expression, which will be explained in following sections. The activated genetic pathways further affect expressions of proteins in both cadherin adhesion and β-catenin degradation modules through genetic feedback loops. The interplay between Wnt signaling and cell adhesion is captured by inter-module crosstalk. Three different kinds of crosstalk are identified, as labeled with numbers in Fig. 1. Here is the overall organization of the method part. Each individual module will be respectively described in details. The crosstalk of different modules will then be summarized. The full mathematical representation of our network model is provided in Document S1.

### Cadherin-mediated cell adhesion

The module of cadherin-mediated cell adhesion describes the dynamic interactions between cadherins and β-catenins during the formation of adherens junctions. The classical cadherins consist of five extracellular domains (EC1-EC5); a single trans-membrane segment; and a highly conserved cytoplasmic domain that contains binding sites for P120-catenin, β-catenin and α-catenin [43], [44]. It is generally believed that the binding of cadherins with β-catenins stabilizes the adherens junctions. Therefore, in current model the cadherin/β-catenin complex (Cad/Cat for short in Table 1 and in the following text) is the basic adhesive unit during cell adhesion. The binding of P120-catenins can protect cadherins from endocytosis [45]. To avoid further complexity of our model, the P120 signaling is not considered and the process of cadherin endocytosis will be introduced in the next paragraph. The α-catenin is thought to regulate the connection between cadherin and actin filaments in a dynamic manner which is also beyond the scope of current study [46].

**Table 1:** State variables and initial conditions of the model.

Network components	Description	Initial Concentration	Initial number of molecules in simulations	References
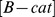	β-catenin in cytoplasm	20 nM	300	Tan et al, 2012
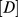	destruction complex	60 nM	900	Choi et al, 2006
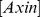	Axin	20 nM	300	Tan et al, 2012
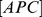	APC	200 nM	3000	Estimated
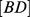	β-catenin bound destruction complex	4 nM	60	Choi et al, 2006
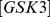	GSK3	200 nM	3000	Tan et al, 2012
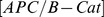	APC/β-catenin complex	0.2 nM	3	Choi et al, 2006
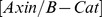	Axin/β-catenin complex	0.06 Nm	1	Choi et al, 2006
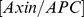	Axin/APC complex	40 nM	600	Estimated
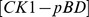	CK1-phospho-β-catenin bound destruction complex	2 nM	30	Lee et al, 2003
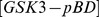	GSK3-phospho-β-catenin bound destruction complex	2 nM	30	Lee et al, 2003
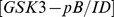	GSK3-phospho-β-catenin bound inactive-destruction complex	6 nM	90	Estimated
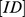	inactive destruction complex	60 nM	900	Estimated
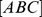	active β-catenin	2 nM	30	Tan et al, 2012
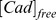	free cadherin on membrane surfaces	E-cad: depends on adhesion condition; N-cad: 0	E-cad: depends on adhesion condition; N-cad: 0	Tan et al, 2012
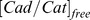	cadherin/β-catenin complex	E-cad: depends on adhesion condition; N-cad: 0	E-cad: depends on adhesion condition; N-cad: 0	Tan et al, 2012
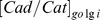	cadherin/β-catenin complex in Golgi	60 nM	900	Estimated
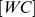	Wnt/receptors complex	0	0	
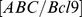	ABC/Bcl9 Complex	0.2 nM	30	Estimated
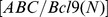	ABC/Bcl9 Complex	0.2 nM	30	Estimated
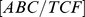	ABC/Bcl9/TCF Complex	0.2 nM	30	Estimated
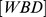	β-catenin/destruction complex bound Wnt/receptor Complex	0	0	
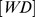	Wnt/receptor bound destruction complex	0	0	
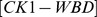	Wnt/receptor bound destruction complex with CK1-phospho-β-catenin	0	0	
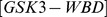	Wnt/receptor bound destruction complex with GSK3-phospho-β-catenin	0	0	
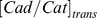	cadherin/β-catenin complex in *trans*-dimer	E-cad: depends on adhesion condition; N-cad: 0	E-cad: depends on adhesion condition; N-cad: 0	
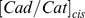	cadherin/β-catenin in *cis*-cluster	E-cad: depends on adhesion condition; N-cad: 0	E-cad: depends on adhesion condition; N-cad: 0	
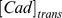	cadherin in *trans*-dimer	E-cad: depends on adhesion condition; N-cad: 0	E-cad: depends on adhesion condition; N-cad: 0	
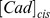	cadherinin *cis*-cluster	E-cad: depends on adhesion condition; N-cad: 0	E-cad: depends on adhesion condition; N-cad: 0	

**Figure 2:**
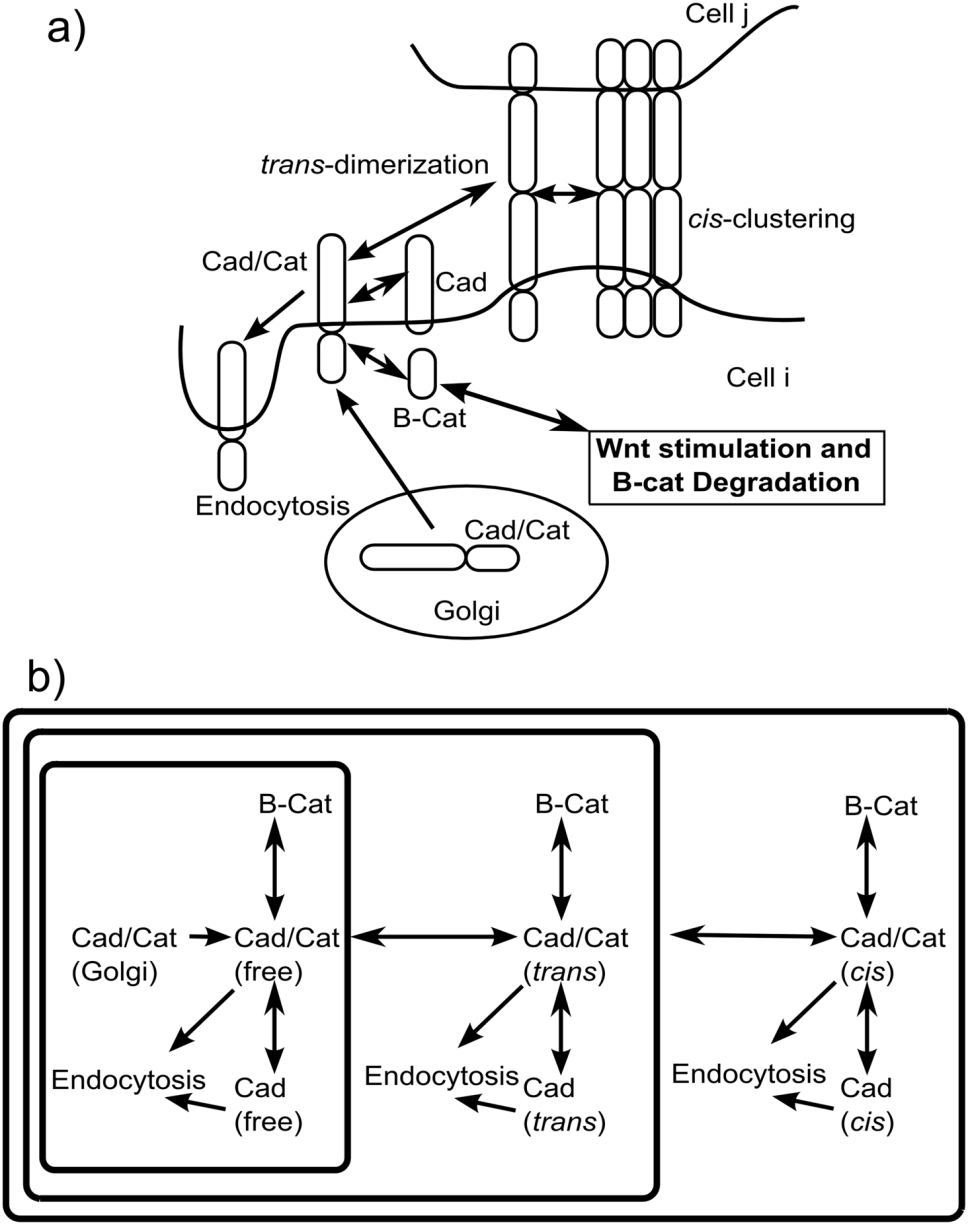
The cadherin-mediated adhesion module. **a)** The Cad/Cat complexes form adherens junctions through a multi-step process. The adhesion is initiated by forming cadherin dimers from apposed cell surfaces through the *trans*-interactions. The clustering of cadherins through the *cis*-interactions triggers the final formation of adherens junctions. **b)** The process of junction formation is simplified by using state transition of Cad/Cat complexes. A “free” Cad/Cat complex can transit to “*trans*” state. Likewise, a “*trans*” Cad/Cat complex can either participate into clusters by transiting to “*cis*” state, or breaks down into “free” state. The endocytosis, association and dissociation of Cad/Cat complexes under different adhesive circumstance are also considered in our model.

The concentration of Cad/Cat complexes on cell surfaces is modulated by three processes (**Fig. 2a**). The first one is the synthesis of Cad/Cat complexes. Previous experiments have demonstrated that Cad/Cat complexes are formed co-translationally in the Endoplasmic Reticulum/Golgi compartments and then shuttle onto cell membrane [47]. In our model, we use a single order chemical reaction to describe this process (reaction 12 in Table 2). The second process is the degradation of Cad/Cat complexes. The rearrangement of Cad/Cat complexes and regulation of their activities on cell surfaces are mediated by cell endocytosis. Cad/Cat complexes are first swallowed into cytoplasm, and then either recycled to cell surfaces or degraded [48]. In our model, recycling is not considered explicitly. We use a single order chemical reaction to describe the degradation (reaction 13 in Table 2). The third process is the dissociation of Cad/Cat complexes. The Cad/Cat complexes are resolved into free membrane-bound cadherins and cytoplasmic β-catenins. The dissociation of Cad/Cat complexes and association of β-catenins and free cadherins reach equilibrium to maintain the stable level of Cad/Cat complexes on cell surfaces [49]. We use a second-order chemical reaction to describe this process (reaction 19 in Table 2).

**Table 2:** Reactions and rate constants in the model.

	Index	Chemical reactions	Descriptions	Parameters
	(1)	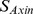	Synthesis of proteins	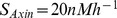
	(2)	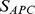		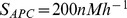
	(3)	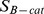		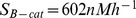
	(4)	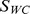		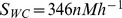
	(5)	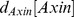	Degradation of proteins	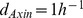
	(6)	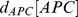		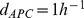
	(7)	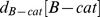		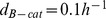
	(8)	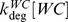		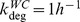
	(9)	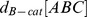		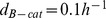
Adhesion Network	(10)	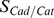	Synthesis of Cad/Cat complex in Golgi	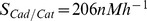
	(11)	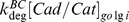	Degradation of Cad/Cat complex in Golgi	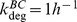
	(12)	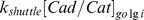	Shuttling Cad/Cat complex from Golgi to cell surfaces	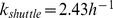
	(13)	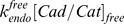	Endocytosis of Cad/Cat complex on cell surfaces	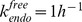
	(14)	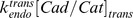		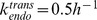
	(15)	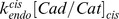		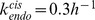
	(16)	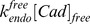	Endocytosis of cadherin on cell surfaces	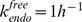
	(17)	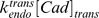		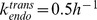
	(18)	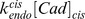		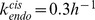
	(19)	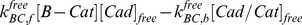	Formation of Cad/Cat complex	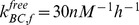 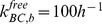
	(20)	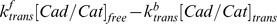		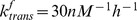 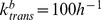
	(21)	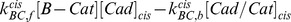		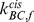 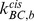 depends on membrane organization effects
	(22)	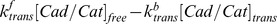	Formation of trans-dimer	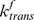 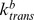 depends on adhesion condition
	(23)	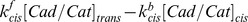	Formation of cis-cluster	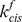 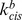 depends on adhesion condition
Destruct Complex Cycle	(24)	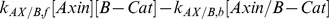	B-cat retention caused by Axin.	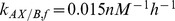 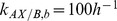
	(25)	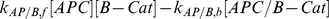	B-cat retention caused by APC.	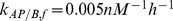 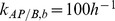
	(26)	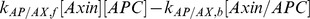	Formation of Axin/APC	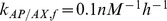 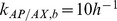
	(27)	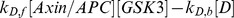	Formation of destruction complex	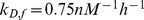 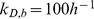
	(28)	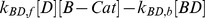	Binding B-cat by destruction Complex	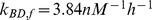 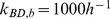
	(29)	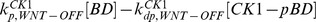	Phosphorylation of B-cat by CK1	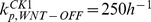 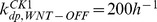
	(30)	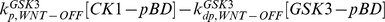	Phosphorylation of B-cat by GSK3	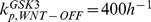 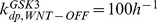
	(31)	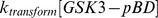	Transformation of Destruction Complex	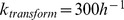
	(32)	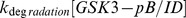	Degradation of GSK3-pB	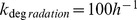
	(33)	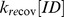	Recovery of Destruction Complex	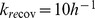
	(34)	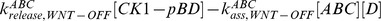	Release of ABC	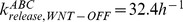 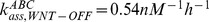
Wnt Activation	(35)	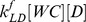	Binding to Wnt/receptor complex	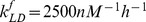
		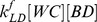		
		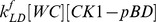		
		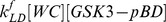		
	(36)	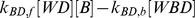	Binding B-cat by destruction Complex	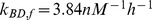 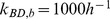
	(37)	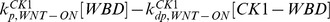	Phosphorylation of B-cat by CK1	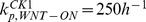 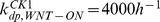
	(38)	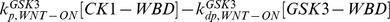	Phosphorylation of B-cat by GSK3	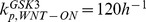 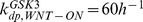
	(39)	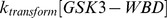	Transformation of destruction complex	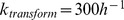
	(40)	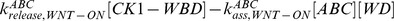	Release of ABC	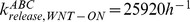 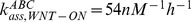
Signaling transduction	(41)	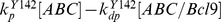	ABC binds to Bcl9	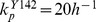 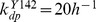
	(42)	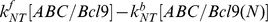	ABC/Bcl9 transports into Nuclear	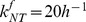 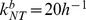
	(43)	 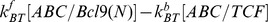	ABC binds to TCF	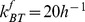 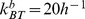

The Cad/Cat complexes further form adherens junctions through a multi-step process (**Fig. 2a**). Before adhesion, randomly dispersed cadherins on originally isolated cells diffuse to the interface region. The adhesion is initiated by forming cadherin dimers from apposed cell surfaces through the *trans*-interactions. The clustering of cadherins through the *cis*-interactions triggers the final formation of adherens junctions [50]–[53]. Other cellular issues might also be involved such as deformations of cell membrane. To avoid introducing further complexity into our model, the process of junction formation is simplified by using state transition of Cad/Cat complexes (**Fig. 2b**). Specifically, Cad/Cat complexes are labeled by three states: “free” Cad/Cat complexes that remain unbound on cell surfaces; “*trans*” Cad/Cat complexes that form trans-dimers with cadherins on the neighboring cells; and “*cis*” Cad/Cat complexes that form *cis* clusters on cell surfaces. A “free” Cad/Cat complex can transit to “trans” state (reaction 22 in Table 2). Likewise, a “*trans*” Cad/Cat complex can either participate into clusters by transiting to “*cis*” state (reaction 23 in Table 2), or breaks down into “free” state. The endocytosis, association and dissociation of Cad/Cat complexes under different adhesive circumstance are also considered in our model (**Fig. 2b**). The rates, however, depend on the state of complexes. Considering that cell adhesion changes the membrane environments and stability, we assume that the endocytosis rate of “*cis*” complexes is lower than that of “*trans*” complexes, and “free” Cad/Cat complexes have the highest endocytosis rate. The association and dissociation rates of “free” complexes equal to that of “*trans*” complexes, but it is different from that of “*cis*” complexes. This is based the fact that *cis*-clustering leads to the spatial organization of Cad/Cat complexes on cell membrane surfaces. The spatial confinement increases the local concentration of Cad/Cat complexes and brings constraints to their diffusions. These further result in the acceleration of associations between cadherins and β-catenins, while their dissociations are slowed down [54], [55].

### Wnt stimulation and β-catenin degradation

**Figure 3:**
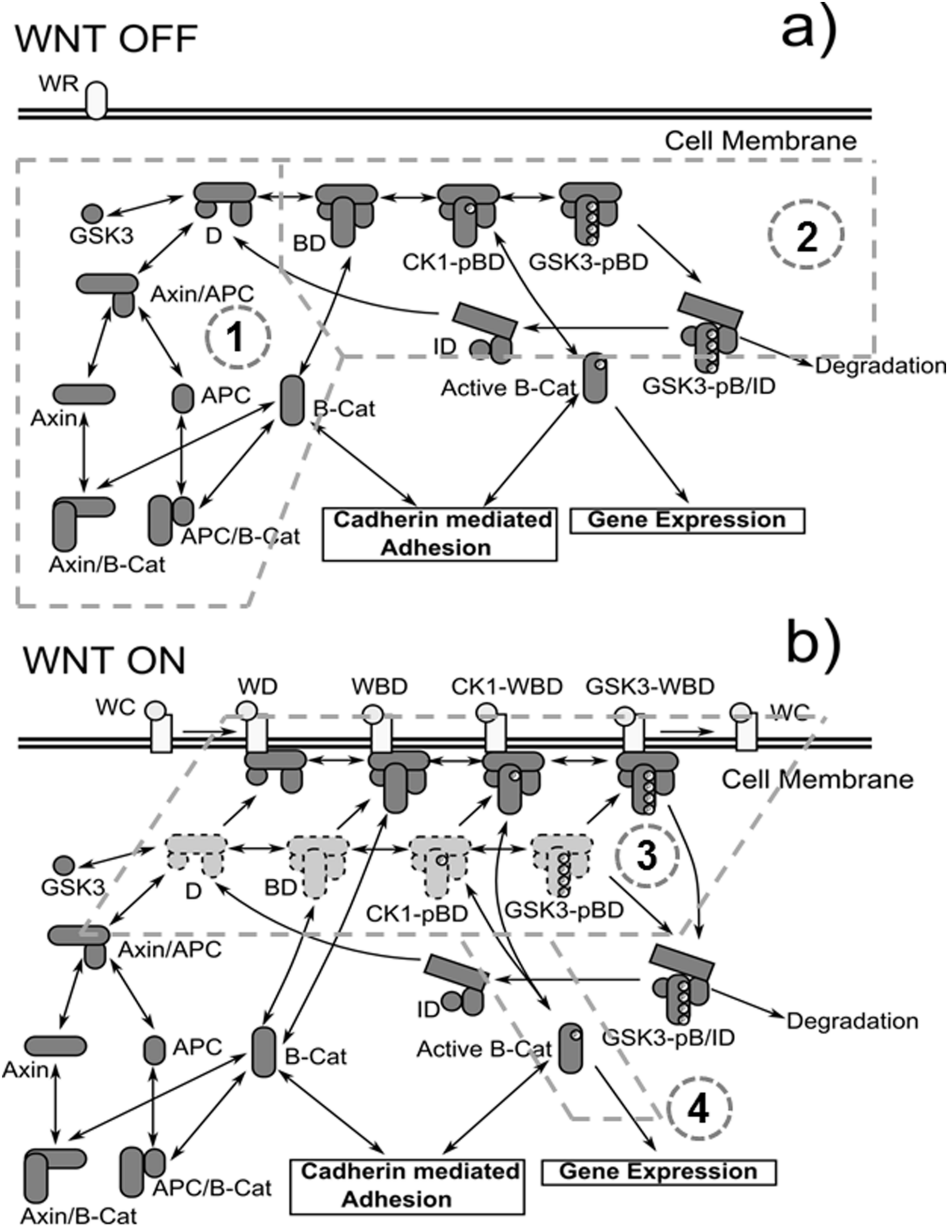
Wnt stimulation and β-catenin degradation module. The module is divided into four parts: **a)** (1) formation of destruction complex and retention of β-catenins by APC and Axin; (2) destruction complex cycle; **b)** (3) Wnt stimulation and (4) Wnt signal transduction.

The module of Wnt stimulation and β-catenin degradation is extended from Lee’s original model and divided into four parts: (1) formation of destruction complex and retention of β-catenins by APC and Axin; (2) destruction complex cycle; (3) Wnt stimulation and (4) Wnt signal transduction. They are labeled with numbers in Fig. 3A and Fig. 3B. The formation of destruction complex and retention of β-catenins in Lee’s model have been validated and supported by experimental results [28]. We inherited this part from Lee’s model. As described in Lee’s model, the formation of destruction complex is initialed by formation of APC/Axin complex. The minimalistic unit of destruction complex (D for short in Table 1 and in the following text) is then formed after GSK3 binds to the APC/Axin complex. The retention of β-catenins is described by formation of APC/β-catenin complexes (APC/B-cat for short in Table 1 and in the following text) and Axin/β-catenin complexes (Axin/B-cat for short in Table 1 and in the following text) separately.

After the formation of destruction complex, we extended the simplified destruction complex cycle in Lee’s model based on recent new experimental evidences. The new destruction complex cycle consists of two parts: destruction complex mediated phosphorylation and degradation of β-catenin after phosphorylation. The phosphorylation of β-catenin has been found to have sequential steps: kinase CK1 first phosphorylates β-catenins at residue S45 (CK1-pBD for short in Table 1 and in the following text). CK1-phosphorylated sites then facilitate downstream phosphorylation at residue S31/37/T41 by GSK3 (GSK3-pBD for short in Table 1 and in the following text) [56]. The degradation of β-catenins after phosphorylation is not clearly understood. Current experimental evidences indicate that releasing β-catenins from destruction complexes is not indispensable for degradation of β-catenin in destruction complex cycle [56]. Structural studies of destruction complexes imply that destruction complex cycle may be mediated by the structural transformation of destruction complexes rather than the complex disassembly [57]. Therefore, we assume that destruction complexes undergo structural transformation that exposes β-catenins to ubiquitin/proteosome machinery after GSK3-phosphorylation. Once β-catenins are degraded, destruct complexes transform back to their initial form and degrade more β-catenins. Furthermore, Kim and coauthors have found that dephosphorylated Axins in destruction complexes has lower binding affinity to LRP6 [25]. Under Wnt treatment, the binding affinity between Axin and LRP6 increases in the first 0.5 hr and then decreases to its initial level after 2 hr. Similar activities have also happened to GSK3-phosphorylated β-catenin level, which decreases in the first 0.5 hr then increase to its initial level after 2 hr under Wnt stimulation [24]. The authors assume that after GSK3 phosphorylation of β-catenins, Axins in destruction complexes gets dephosphorylated and becomes “inactive” (GSK3-pB/ID for short in Table 1 and in the following text). Nusse and colleagues have demonstrated that β-catenins could release from dephosphorylated Axins [58]. Therefore, we further assume that the dephosphorylation of Axins trigger the transformation of destruction complex which leads to the exposure of β-catenins to ubiquitin/proteosome machinery.

Base on the assumptions above, we describe the new destruction complex cycle as follows (**Fig. 3a**): destruction complexes (D) first bind to β-catenins (BD for short in Table 1 and in the following text). CK1s then phosphorylate β-catenins in destruction complexes which generate CK1-pBD complexes (reaction 29 in Table 2). CK1-pBD complexes further transits into GSK3-pBD by GSK3-mediated phosphorylation (reaction 30 in Table 2). After that, Axins get dephosphorylated, and GSK3-pBD complexes transform into GSK3-pB/ID complexes which expose β-catenins to ubiquitin/proteosome machinery (reaction 32 in Table 2). When β-catenins are degraded, GSK3-pB/ID complexes turn to “inactive” destruction complexes (ID for short in Table 1 and in the following text) (reaction 31 in Table 2). Axins in the “inactive” destruction complexes can get phosphorylated again so that “inactive” destruction complexes (ID) transform back into active destruction complex (D) to degrade more β-catenins (reaction 33 in Table 2).

After Wnt stimulation, it was found that receptors like LRP6 and Frizzled bind together to the extracellular Wnt target proteins. LRP6 receptors then get phosphorylated and recruit Axin onto membrane proximal regions [9], [16]. According to the recent experimental results, the destruction complex still remains active under wnt stimulation [23]. In our model, the destruction complex cycle we described in the last paragraph is functional in “Wnt off” scene (**Fig. 3a**). In “Wnt on” scene, it is split into two conjunctive cycles with one in cytoplasm (cytoplasmic cycle) and the other on membrane surface (membrane cycle) (**Fig. 3b**). All intermediate states in the cytoplasmic cycle have a corresponding counterpart in the membrane cycle and they can transfer to the membrane surface from cytoplasm (reaction 35 in Table 2). The cytoplasmic cycle in “Wnt off” scene still remains active under Wnt stimulation, but it is no longer the major pathway for destruction activities. In “Wnt on” scene, majority of destruction complexes constructed by phosphorylated Axin are recruited onto membrane proximal regions and bind to Wnt receptors. In the membrane cycle, the phosphorylation rates of β-catenins decrease to a lower level (reaction 37 and 38 in Table 2) [24]. After phosphorylation of β-catenins, Axins are dephosphorylated. This generates GSK3-pB/ID complexes, which are then released from Wnt receptors (reaction 39 in Table 2). Finally, the cytoplasmic and membrane cycles merge into the same pathway, as shown in **Fig. 3b**.

The Wnt signal transduction pathway was also updated. H. Clever and colleagues have proved that only the N-tail dephosphorylated β-catenins from destruction complex (so called Active β-catenins) could trigger Wnt target gene expressions [59]. Recent experiments have also proved that CK1-phosphorylated β-catenins are quite similar to the active β-catenins (ABC for short in Table 1 and in the following text) [24]. We assume ABC could release from the destruct complex cycle at the stage of CK1-pBD (reaction 40 in Table 2). Under Wnt stimulation, CK1-pBD binds to Wnt receptors (CK1-WBD for short in Table 1 and in the following text) to form complexes. These complexes were proposed to trigger structural changes that facilitate the release of ABC. Therefore, we further assume that ABC release from CK1-pBD in “Wnt off” scene with much lower rate than that in “Wnt on” scene. After releasing from destruction complex, ABC may participate in cell adhesion after binding to cadherins, or participate in downstream signaling after entering cell nuclear, or reenter the destruction cycle. Experimental studies have demonstrated that binding to Bcl9 is a functional switch for β-catenins between cell adhesion and Wnt signaling. This functional switch can be modified by phosphorylation of β-catenins at residue Y142 [60]. More details about this functional switch will be introduced in the following sections.

### Wnt target gene expressions

The module of gene expression describes the transcriptional regulations of Wnt target genes. After an active β-catenin binds to Bcl9, the dimer (ABC/Bcl9 for short in Table 1 and in the following text) shuttles into cell nucleus (reaction 42 in Table 2). The nuclear ABC/Bcl9 (ABC/Bcl9(N) for short in Table 1 and in the following text) further forms a complex with TCF (ABC/TCF for short in Table 1 and in the following text) in cell nucleus (reaction 43 in Table 2) and activates the expressions of Wnt target genes [9], [16]. Because hundreds of genes have been identified as targets for Wnt signaling under different cell stages and cell lines, it is difficult to construct a generic network to describe the detailed procedure of transcriptional regulations and overall expressions of all Wnt target genes.

**Figure 4:**
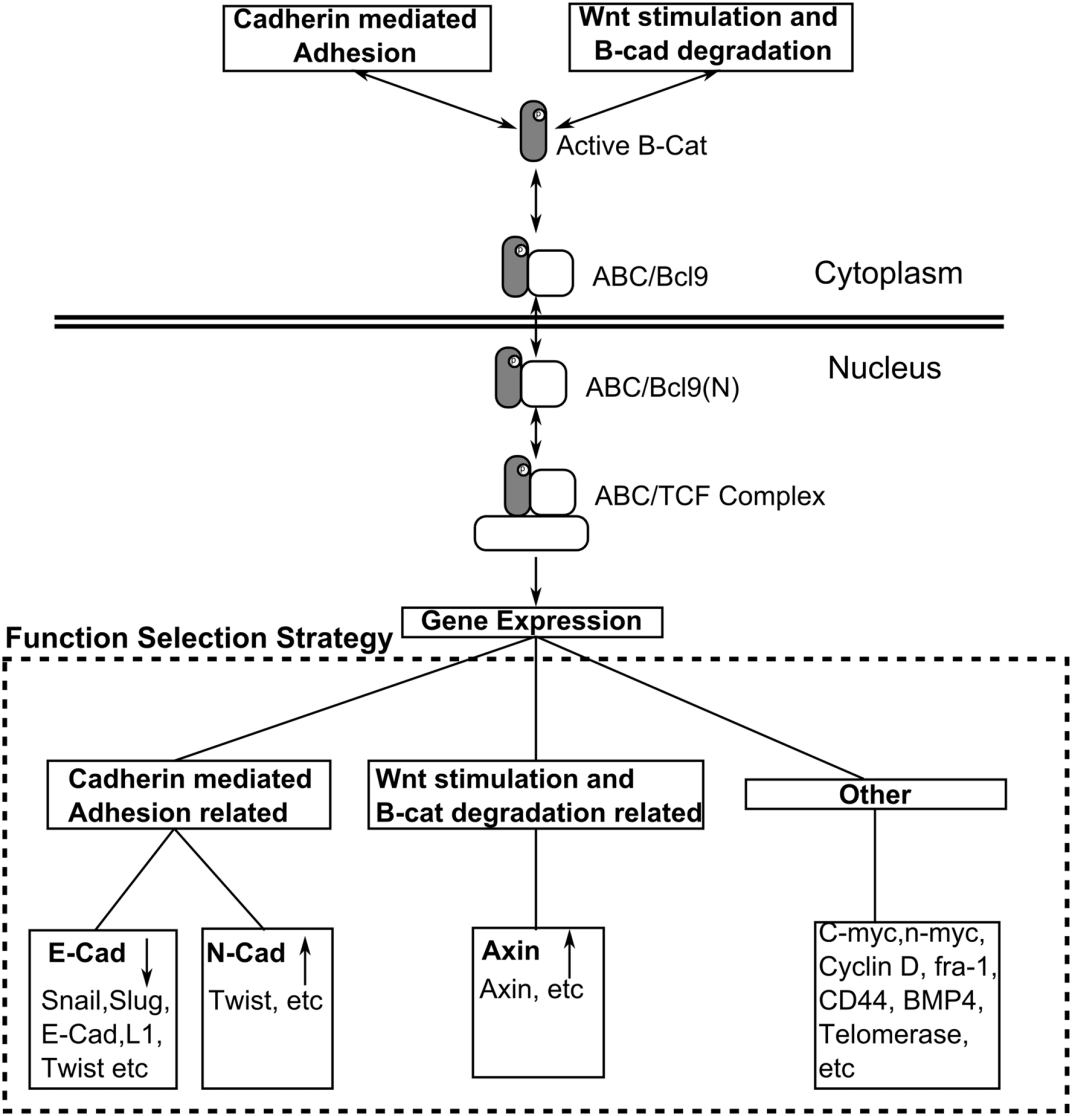
The module of Wnt target gene expressions. The nuclear ABC/Bcl9 further forms a complex with TCF in cell nucleus and activates the expressions of Wnt target genes. We applied a function selection strategy to allocate the expressions of different Wnt target genes. We divided target genes into three categories by their biological functions. Selections of target genes in each category lead to changes of transcription rates for the corresponding proteins in the network.

To overcome this obscure, we applied a function selection strategy to allocate the expressions of different Wnt target genes and adjust their transcriptional feedback to the cadherin-mediated adhesion and Wnt stimulation modules. We divided target genes into three categories by their biological functions. Selections of target genes in each category lead to changes of transcription rates for the corresponding proteins in the network (Fig. 4). The first category of target genes is related to cadherin-mediated adhesion, such as Slug, Snail and Twist. They change the expression rates of E-cadherin or N-cadherin. The second category of target genes is related to Wnt stimulation and β-catenin degradation. For example, the expression rate of Axin was found to increase under Wnt stimulation. Therefore, Axin is one of the corresponding functional proteins in this category. The last category of target genes participates in signaling events that are beyond the scope of current model, so no functional protein in the network is assigned to this category.

We assume the accumulation of ABC/Bcl9 complexes in cell nucleus and random distributions of ABC/TCF complexes on transcriptional regions of target genes. Consequently, if the concentration of nuclear ABC/Bcl9 complexes is below certain threshold, its effect on changing target gene expressions remains undetectable. Once the nuclear ABC/Bcl9 concentration steps over the threshold, it starts to change the expression levels of Wnt target genes. The expressions are regulated by applying an adjusting factor to the original transcriptional rates of target proteins. The adjusting factor is different for each different target protein. For a specific protein, the transcriptional regulation can be either positive or negative, as shown in Fig. 4. The values of adjusting factors also depend on the relative amount of ABC/TCF in three categories. Following the function selection strategy, each newly formed ABC/TCF complex has a probability to select one of the three categories. After the generation of a new complex or dissociation of an existing complex, the distributions of ABC/TCF in all categories are modified. This further changes the value of the adjusting factor in each category. As a result, the adjusting factor for a specific category i can be written by:
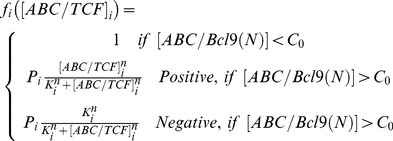
(1)


In equation (1), 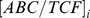 is the amount of ABC/TCF complexes distributed in category i. P_i_ is the probability that a newly formed ABC/TCF complex select the i^th^ category. C_0_ is the threshold above which ABC/TCF complexes start to change the expression levels of Wnt target genes. K_i_ is a constant in the Hill equation to adjust the strength of transcriptional regulation for protein I, and n is the Hill coefficient equals to 7 in current model. The full descriptions of adjusting factors in the gene expression module are listed in Table 3. Particularly for E-cadherin, because its expression also co-regulated by GSK3, its negative adjusting function is further given by:

**Table 3:** The adjusting factors and parameters in the module of gene expression.

Targets	Equations	Parameters
E-Cadherin	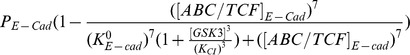	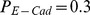 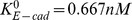 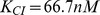
N-Cadherin	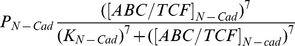	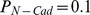 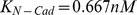
Axin	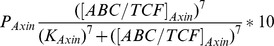	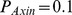 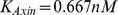
Inhibitor 2	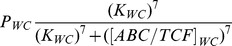	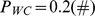 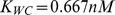
Other	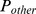	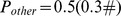



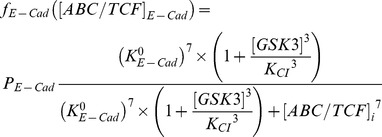
(2)The 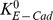 in equation (2) is the regulation constant for E-cadherin without GSK3, and K_CI_ is the coefficient to determine the extent of cooperative inhibition from GSK3. Finally, transcription feedback loops were integrated into the model by multiplying these adjusting factors to the original synthesis rates of target proteins in the network, as shown in equation S1 for cadherin and equation S8 for Axin in Document S1.

### Interplay between network modules

The interplay between Wnt signaling and cell adhesion is captured by inter-module crosstalk. Newly generated ABCs that are released from destruction complexes can bind cadherins to form Cad/Cat complexes, be phosphorylated to trigger the target gene expressions, or bind to destruction complexes again and be degraded by proteasome. Thus, the cell fate after Wnt stimulation is determined by the co-regulation of ABC between different modules. Except for the transcriptional feedback loops introduced in the last section, in which the network components in both adhesion and degradation modules are regulated by the activation of genetic pathways, three additional types of crosstalk are further proposed here. As labeled with numbers in Fig. 1, cadherin mediated adhesion can: (1) compete for ABCs with their degradation; (2) affect the kinetics of destruction cycle through membrane spatial organization; and (3) modulate phosphorylation rate of ABCs and their binding with Bcl9.

The β-catenins can competitively bind to both cadherins and destruction complexes. The competition of cell adhesion for ABCs is regulated by the concentrations of cadherins and their interactions with β-catenins. The concentrations of cadherins on cell surfaces depend on the states of cell adhesion. It was found that the concentrations of Cad/Cat complexes increase under strong adhesion [48]. Therefore, in our model, the total amount of Cad/Cat complexes in “*cis*” state is larger than that in “*trans*” state, while the total amount of Cad/Cat complexes in “*trans*” state is further larger than that in “free” state. Moreover, cell membrane surfaces are spatially reorganized during cadherin clustering. Previous studies indicate that the formation of signaling platform during receptor clustering facilitate the binding between receptors and intracellular scaffold proteins. Therefore, we assume the spatial reorganization of membrane changes the binding between cadherins and β-catenins. The association rate between cadherins and β-catenins increases and the disassociation rate decreases. The quantitative evaluation and the functional impacts of these changes will be discussed in the results section.

In addition to the competition for ABCs, cadherin-mediated cell adhesion also actively affects the kinetics of destruction cycle. Maher et al have demonstrated that destruction complexes gather around adhesive junctions near cell surfaces [14]. The kinetics of β-catenin degradation in the membrane-associated destruction complex is enhanced by adhesion. Because the spatial reorganization of cell membrane during adhesion generates a crowding environment for destruction complexes, it is a possible mechanism that this reorganization affects the destruction complex cycle. As mentioned earlier, destruction complexes undergo a structural transformation and expose β-catenins to ubiquitin/proteasome machinery. Previous studies have shown that crowding environments inhibit unfolding of proteins [61]. Similarly, we assume that the crowding caused by cadherin clustering slows down the structural transformation in destruction complexes, which leads to more ABCs released. Besides the structural transformation of destruction complex, the binding between destruction complexes and β-catenins and the release of ABCs from destruction complexes are also affected by the crowding environment. We will discuss more about this in result section.

Thirdly, there are specific sites of phosphorylation in newly generated ABCs facilitating Wnt signaling. For example, phosphorylation of β-catenins at residue Y142 by the kinase c-Met promotes Bcl9 binding, therefore acts like a functional switch between cadherin-mediated adhesion and gene activation [1]. The kinase c-Met and most other kinases that phosphorylate β-catenins are either membrane receptors or membrane-associate proteins. Therefore, their activities are also influenced by membrane reorganization caused by cadherin clustering. The phosphorylation can be affected either positively or negatively. It is possible that the spatial organization of Cad/Cat complexes enhances the encounter rate between kinases and ABCs and promotes the phosphorylation. Detailed discussions are presented in result section.

In summary, the abbreviations of all the molecular components in the network are presented in Table 1. Table 2 gives all the reactions between molecules and the values of their reaction parameters with brief descriptions. Table 3 gives all the adjusting factors used in the gene expression module. The full mathematical representation of our model can be found in Document S1 (from equation S1 to equation S25). Finally, the dynamics of the network was stochastically simulated with the Gillespie algorithm based on the given equations.

## Results

### Calibration of Wnt stimulation and β-catenin degradation module

As introduced in the method, the Wnt stimulation and β-catenin degradation module in our network incorporated features from various existing models. Before applying this module to investigate the interplay between cadherin-mediated adhesion and Wnt signaling, the parameters such as initial concentration of each molecule and rate constants of each reaction need to be calibrated so that recent experimental results can be qualitatively reproduced by the module. To minimize the complexity during the calibration, we eliminated any effect that is potentially caused by cell adhesion. Consequently, the calibration was performed to single cells in which the formation of *trans*-dimers and *cis*-clusters were inhibited.

It should be pointed out that the parameters of network components vary from different cell lines and cell stages. It is difficult to acquire a whole set of parameters that are consistent with all cell lines or cell stages. Therefore, the values of molecular concentrations were chosen from the range of different experimental measurements. For an example, the measured concentrations of ABC in cytoplasm range from 1 nM to 261 nM for different cell lines. As a result, the initial concentration of cytoplasmic ABC in our model is set to 2 nM. Furthermore, the concentrations were converted to the molecular numbers during stochastic simulations. We set up a subcellular system that occupies 2.5% volume of a whole cell. Given the measurement that the volume of a cell is around 10^−12 ^L, and the initial concentration of cytoplasmic ABC is 2 nM, the system includes 30 free ABC molecules in the beginning of simulations. The detailed initial values of other molecules in the module are listed in Table 1.

Comparing with the initial concentrations, much less experimental information about rate constants is available in literature. Choi and colleagues measured the binding affinity between β-catenins and different proteins such as APC and E-cadherins. The affinity is used to determine the ratio between on and off rates of corresponding reactions [62]. The specific values of the kinetic parameters were adopted from the typical range of chemical reactions and were manually optimized by reproducing the following experimental results. 1) Under continuous Wnt stimulation, the total β-catenins increase to six fold of its initial level; 2) the increase of non-phosphorylated β-catenins is comparable to that of β-catenins; 3) the GSK3-phosphorylated β-catenins decrease in 0.5 hr then recover to its initial level in 2 hr [24]; 4) the CK1-phosphorylated β-catenins decrease in 0.25 hr then increase to 3 fold of its initial level in 2 hr; and 4) the binding affinity between Axin and LRP6 increases in 0.5 hr then decreases in 2 hr [25].

**Figure 5:**
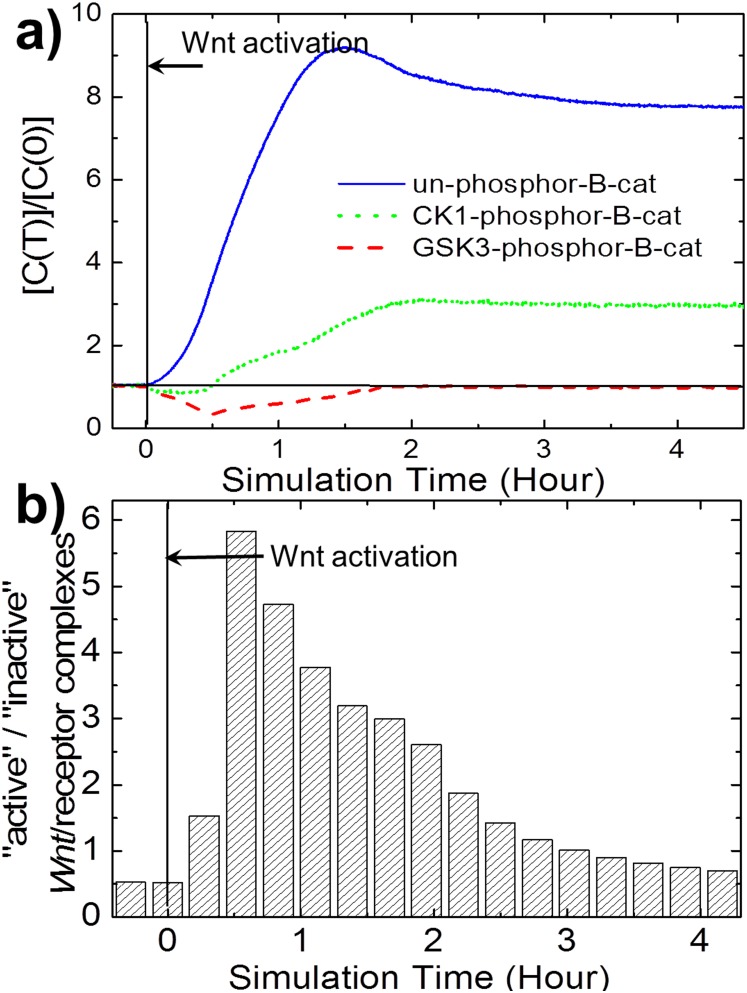
Calibration of Wnt stimulation and β-catenin degradation module. **Fig. 5a** shows the kinetic respond of β-catenins. GSK3-phosphor-B-cat (red dash line) decreases in the first 0.5 hr then recovers to its initial level. CK1-phosphor-B-cat (green dot line) decreases in the first 0.5 hr then increases to around 3 fold of its initial level. Un-phosphorylated B-cat (blue solid line) increases to 8 fold of its initial level. **Fig. 5b** shows the kinetic response of Axin-bound Wnt/receptor complexes to Wnt stimulation. All curves were averaged over 50 simulation trajectories. Our model reproduced the most recent experimental phenomena with semi-quantitative accuracy.

As shown in Fig. 5a, our simulation results indicate that the calibrated parameters have qualitatively reproduced the kinetic responds of β-catenins to Wnt stimulation. Free non-phosphorylated β-catenins in cytoplasm increases to 8 fold of its initial level after 4 hr (**Fig. 5a** blue solid line). The GSK3-phosphorylated β-catenins decrease in the first 0.5 hr, but recover to its level in 2 hr (**Fig. 5a** red dash line). The CK1-phosphorylated β-catenins first decrease then increase to 3 fold of its initial level in 2 hr (**Fig. 5a** green dot line). Additionally, as described in Kim’s model, phosphorylated Axins by GSK3 have stronger binding affinity to LRP6 than dephosphorylated Axins. We labeled Wnt/receptor complexes that contain phosphorylated Axins as “active”, and those contain dephosphorylated Axins as “Inactive” in our simulations. The results demonstrated that the ratio between “active” and “inactive” Wnt/receptor complexes in 0.5 hr and decrease after 2 hr under Wnt stimulation (**Fig. 5b**). This indicates that the binding affinity between Axin and LRP6 increases in 0.5 hr and decreases after 2 hr, which is consistent with the experimental observation. Taken together, the Wnt stimulation and β-catenin degradation module reproduced the most recent experimental phenomena with semi-quantitative accuracy. The calibrated module provides a robust basis to investigate the interplay between cadherin mediated adhesion and Wnt signaling.

### The competition of β-catenins between Wnt signaling and cadherin-mediated adhesion

The ABCs released from destruction complexes can either bind to free cadherins on cell surfaces to stabilize adhesive junctions or re-associate with destruction complexes before degradation. The competitive binding of β-catenins to cadherins against destruction complexes is the most straightforward interplay between these two systems. As cell adhesion becomes stronger, the amount of Cad/Cat complexes on membrane surfaces increases. To reduce complexity, we first neglected the effects of membrane organization under cell adhesion. As a result, the binding affinity between cadherins and β-catenins remains unchanged. Given the same binding affinity between cadherins and β-catenins, the amount of free cadherins on membrane surfaces also increases, resulting in the fact that more ABCs are recruited onto membrane surfaces and form Cad/Cat complexes. Therefore, the balance of ABCs’ cytoplasmic distributions is broken by the change of adhesive conditions.

In our model, the adhesive condition of cells are described by the ratio of cadherins in “free”, “*trans*” and “*cis*” states when equilibrium is reached. For examples, the ratio of three states for an isolated cell is 1∶0∶0, because there is no adhesion junction on its surfaces. For cells only expressing mutated cadherins that cannot form cis interactions, the ratio is 1:X:0 because there is no cadherin in “*cis*” state. Ratios for wild type cells are 1:X:Y and the values of X and Y/X are related to the stability of the adhesive junction and the binding affinity of *trans* and *cis* interactions. In our simulations, the specific values of X and Y/X after equilibrium were archived by adjusting the transition rates of Cad/Cat complexes between different adhesive states, as indicated in Table 2.

**Figure 6:**
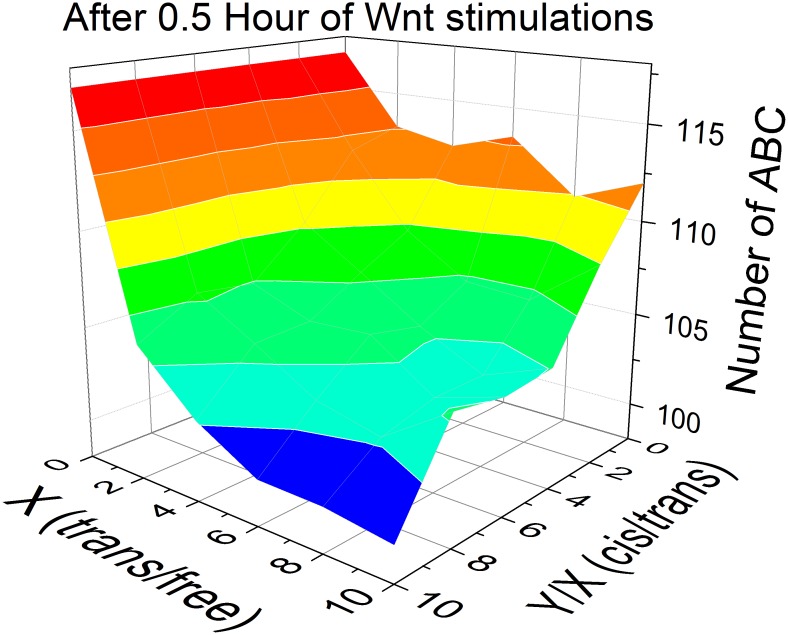
The 2D phase diagram gives the number of ABC in cytoplasm under different adhesion condition after 0.5 hr Wnt treatment. The diagram is plotted along the ratio between numbers of Cad/Cat in “*cis*” and “*trans*” statues (Y/X), and the ratio between number of Cad/Cat in “*trans*” and “free” status (X). The value of each point is the average of 50 simulation trajectories. The figure illustrates that cell adhesion negatively contributes to Wnt signaling through competition for cytoplasmic ABCs.

In details, multiple values of X and Y/X were chosen to investigate the effects of competition within a wide range of adhesive conditions. The ratios of 1:X:Y are ranged from 1∶0∶0 to 1∶10∶100. For each ratio, the average amount of free ABCs left in cytoplasm after 0.5 hr of Wnt treatment was calculated from 50 simulations trajectories. The simulation results for different ratios of X and Y are plotted as a two-dimensional profile (**Fig. 6**). The figure shows that the amount of free ABCs decreases when the strength of cell adhesion (the ratio of cadherins in "*trans*" versus "free" state) increases. The amount of ABCs further decreases when *cis*-clstering is taken into account, represented by the value of Y/X that increases from 0 to 10. In another word, after forming adherens junctions, more ABCs are recruited to cell surfaces and Wnt signaling pathway is weaken. Therefore, our model illustrates that cell adhesion negatively contributes to Wnt signaling through competition for cytoplasmic ABCs. More importantly, our simulations gave a quantitative estimation of how the amount of ABCs in cytoplasm is related to the strength of *trans* and *cis* interactions.

### Kinetic changes of β-catenin degradation and Wnt signaling induced by cell adhesion

Competition for β-catenins is not the only connection between cell adhesion and Wnt signaling. Maher et al demonstrated that cadherin-based cell adhesion can enhance the kinetics of β-catenin degradation in membrane-associated destruction complexes [14]. Howard et al also showed evidences that cadherins are actively involved in Wnt signaling [15]. Here we proposed that the spatial organizations of cadherin clusters during cell adhesion generate a crowding environment for proteins at membrane proximal regions. Previous studies indicated that clustering prevents long-distance random diffusion of individual receptors in the crowded cellular environment, drives scaffold proteins to form a platform on which further molecules are recruited to trigger downstream signaling cascades [63], [64]. It is possible that cadherin-mediated cell adhesion regulates Wnt signaling processes by similar mechanisms.

We hypothesized that membrane surfaces are divided into “interface” and “non-interface” regions. Cellular junctions are formed at “interface” regions. Therefore, only the reactions at “interface” regions are changed due to spatial organizations. In additional to the slower structural transformation of destruction complex as explained in the method, it was found that protein association is accelerated and dissociation is decelerated in a crowded environment [54], [65]. Here we introduce two parameters that were used to adjust rate constants of “interface” regions. α_1_ (α_1_>1) is a coefficient to describe the increase of rates, including the acceleration of protein-protein association and phosphorylation, while α_2_ (α_2_<1) is a coefficient to describe the decrease of rates, including the deceleration of protein-protein dissociation and structural transformation of destruction complexes in crowding environments. Assuming that corresponding proteins and complexes are uniformly distributed on cell surfaces, the average effects caused by adhesion can be derived by calculating the proportion of Cad/Cat complexes under “*cis*” state (P*_cis_*), where 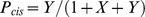. Therefore, the average rates of reactions that become faster are 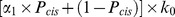, in which k_0_ in the original rate in “non-interface” regions. Similarly, the average rates of reactions that become slower are 
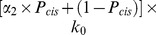
.

**Figure 7:**
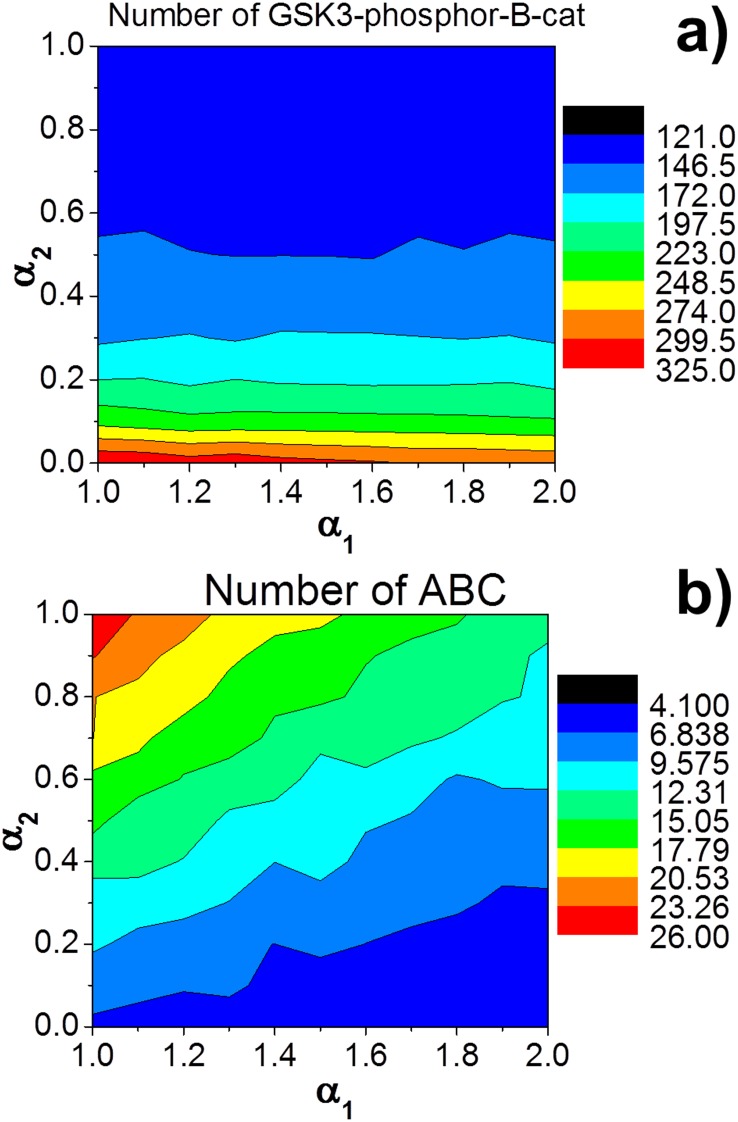
We proposed that the spatial organizations of cadherin clusters during cell adhesion generate a crowding environment for proteins at membrane proximal regions. We introduced two parameters, α1 and α2, to adjust rate constants that are changed by cell adhesion. Without Wnt stimulation, we first studied the changes of β-catenin distributions induced by adhesion. The 2D phase diagram are plotted using different values of α1 and α2, with α1 ranged from 1 to 2 and α2 ranged from 0 to 1. Our results suggest that cell adhesion promote the amount of GSK3-phosphorylated β-catenins **a)**, and reduces the amount of ABC in cytoplasm **b)**.

We first investigated the adhesion-induced changes of β-catenin distributions when there is no Wnt stimulation. Maher et al have found that without Wnt stimulations, the cytoplasmic level of ABC decreases under strong adhesive condition, but the level of GSK3-phosphorylated β-catenins increases. We assume that majority of cadherins form *trans*-dimers due to the diffusion trap mechanism. At cellular interface, majority of *trans-*dimers further form *cis*-clusters. As a result, the ratio X and Y were set to be 10 and 100, respectively. This gives. 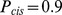 Multiple simulations were carried out by using different values of α1 and α2, so that the effects of cell adhesion can be systematically estimated. Cell adhesion causes no effect to Wnt signaling if both α1 and α2 equal 1. The simulation results are plotted in Fig7, with α1 ranged from 1 to 2 and α2 ranged from 0 to 1. Our results show that cell adhesion reduces the amount of ABC in cytoplasm (**Fig. 7b**) and promote the amount of GSK3-phosphorylated β-catenins (**Fig. 7a**). Therefore, the simulations are consistent with the experimental observations.

**Figure 8:**
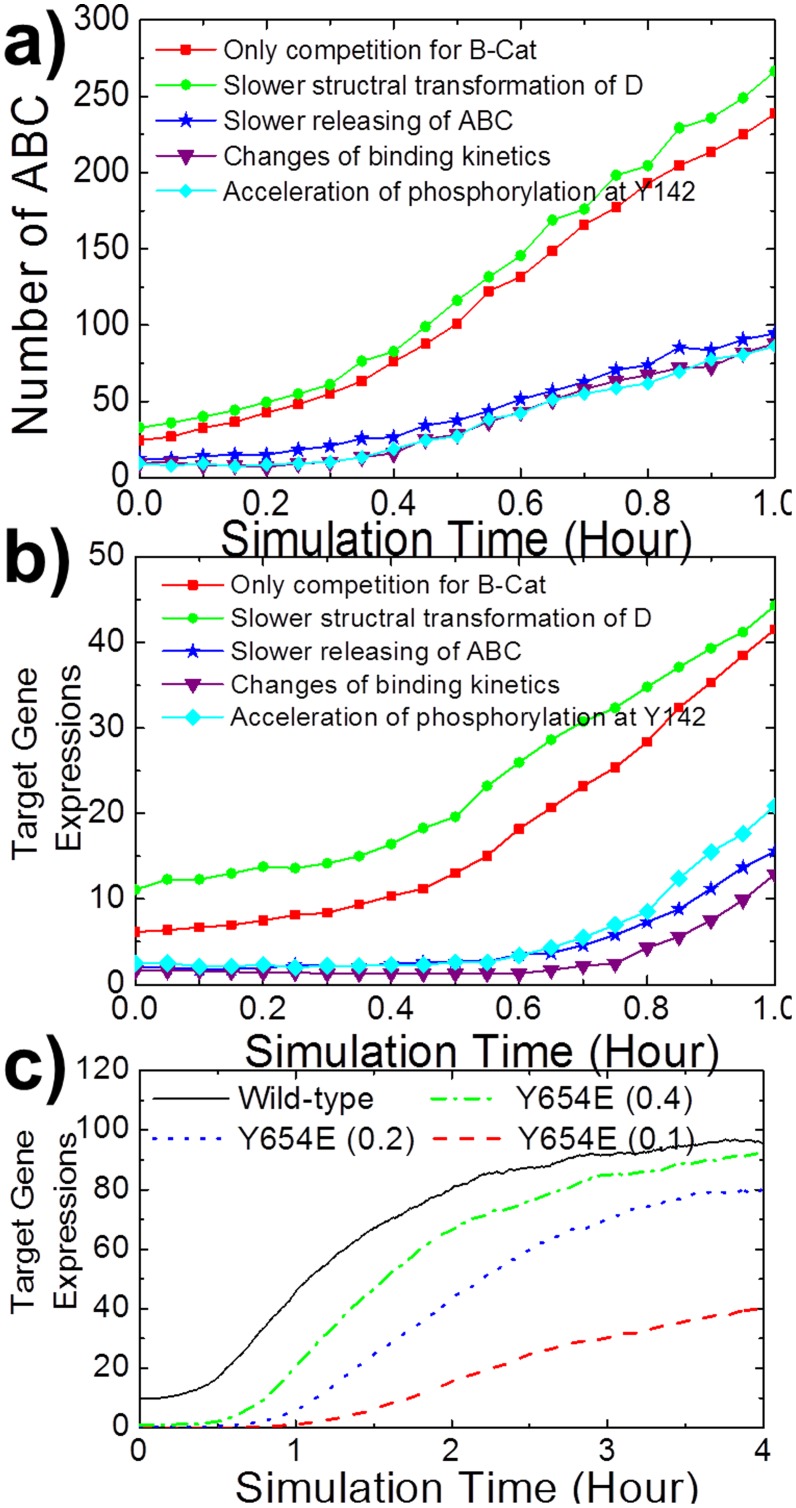
Cell adhesion changes β-catenin distributions under Wnt stimulations. The values α2 = 0.4 and α1 = 1.5 were chosen. In order to minimize the complexity, we decomposed the multiple factors caused by adhesion and added them step by step. The number of ABC and activated target genes are shown in **a)** and **b)**. We suggest that slow releasing of ABCs from destruction complexes is the major factor cause by cell adhesion. We further proposed that cell adhesion also affects Wnt signaling by regulating the phosphorylation rate of β-catenins. Given the truth that the mutant of Y654 reduces the binding affinity between cadherin and β-catenin and decrease the phosphorylation rate of Y142, we set the Y142 phosphorylation rate of Y654 mutant equals to 0.4, 0.2 and 0.1 of the original value in wild-type β-catenin **c)**. We found that the Wnt target gene expressions of Y654 mutants are reduced. All curves were averaged over 50 simulation trajectories.

We further explored how adhesion changes β-catenin distributions under Wnt stimulations. The values α2 = 0.4 and α1 = 1.5 were chosen to quantitatively reflect the experimental data in Maher’s study. In order to minimize the complexity, we decomposed the multiple factors caused by adhesion and added them step by step. Firstly, the red squares in **Fig. 8a** and **Fig. 8b** are the simulation results assuming cell adhesion affects Wnt signaling only through competition. The results indicates that the cytoplasmic concentration of ABC increases to 10 folds after 1 hr of constant Wnt stimulations, and target genes are correspondingly activated. Based on these results, we gradually added more factors. We found that slowing down of destruction complex structural transformation increases the accumulation rate of ABC (**Fig. 8a** green circles). Moreover, reducing the release rate of ABC from destruction complex dramatically decreases the accumulation of ABC (**Fig. 8a** dark blue stars), while changes of binding kinetics between proteins in the network further slightly reduce the accumulation rate of ABC after Wnt stimulations (**Fig. 8a** purple triangles). Therefore, the simulation results suggest that overall strong adhesion negative affect Wnt signaling additional to the simple competition of β-catenins. Slow releasing of ABC from destruction complex plays the major role.

Cell adhesion also affects Wnt signaling by regulating the phosphorylation rate of β-catenins. Brembeck et al have found that the functional switch of β-catenin between adhesion and Wnt signaling is modulated by its phosphorylation status at residue Y142. The kinases related to this phosphorylation are membrane associated receptors which locations are close to cellular junctions. Therefore, we assume that cell adhesion facilitates the phosphorylation of β-catenin at Y142 and the phosphorylation accelerates the association kinetics between ABC and Bcl9. Consequently, the simulation results show that although the acceleration of Y142 phosphorylation does not change the kinetic response of ABC (**Fig. 8a** light blue diamonds), it remarkably affect the equilibrium concentration of ABC and ABC/Bcl9 complexes, therefore increase the target gene expression (**Fig. 8b** light blue diamonds). To further illustrate the functional role of cell adhesion in Y142 phosphorylation, we studied the mutation at residue Y654 of β-catenin that reduces its binding affinity with cadherin. We found that the Wnt target gene expressions of Y654 mutants are reduced (**Fig. 8c**). This is caused by the fact that the low affinity prevents the mutants from entering the junctions, which in turn reduces the Y142 phosphorylation rate related to Bcl9 binding and Wnt activations. It was found that Y654 mutation weakens the gene expression of Wnt target gene. Our model therefore provides the mechanistic interpretation to this phenomenon.

In summary, our computational studies are consistent with various recently experimental data about interplay between cell adhesion and Wnt signaling. In additional to the simple competition for β-catenins, we provide the theoretical basis of how spatial organization of cell adhesion actively regulates the degradation pathway and phosphorylation rate of β-catenins. We suggest that slow releasing of ABCs from destruction complexes is the major factor cause by cell adhesion, which gives potential insights to modulate the Wnt signaling pathway under strong cell adhesion.

### Transcriptional feedback from Wnt target gene expressions

Activation of Wnt target genes changes the transcription rates of proteins that participate in cell adhesion and β-catenin degradation. This provides an additional interplay between cell adhesion and Wnt signaling pathway. For examples, after Wnt stimulations, the transcription rates of N-cadherin increase, while the transcription rates of E-cadherin decrease. Wnt target genes such as Snail and Slug have been identified to inhibit transcription of E-cadherins, while others such as Twist are able to activate transcription of N-cadherins. The expression of E-cadherins is also influenced by GSK3, because GSK3 was found to inhibit the activities of Snail. The transcriptional regulations of N- and E-cadherins lead to the process called “cadherin switch”, in which cells are switched from expression of E-cadherins to expression of N-cadherins. The cadherin switch is one of the most important hallmarks of EMT.

**Figure 9:**
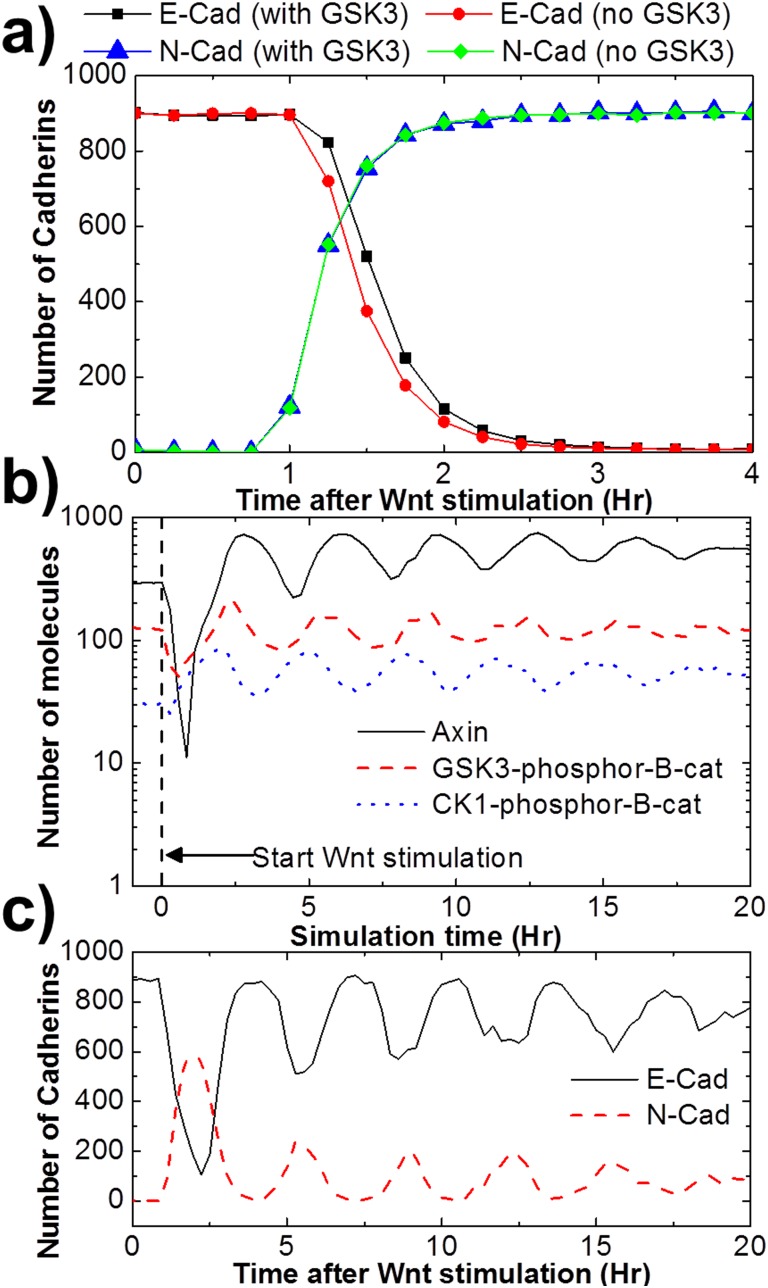
The transcription feedback caused by Wnt signaling. The figure shows **a)** E-N switch of cadherins, and **b)** oscillation of destruction complex cycle. We have further revealed a decayed oscillation during cadherin switch **c)**.

The process of cadherin switch is numerically archived after adding the module of gene expression. As listed in Table 3, we applied Hill equation to describe the positive and negative changes in expression of N- and E-cadherin, and further used competitive inhibition model to consider the effect of GSK3. As a result, the simulation result shows that the concentration of E-Cadherin in Golgi decays after 1 hr delay of Wnt stimulation, while the concentration of N-cadherin increases. The E- and N-cadherins are fully switched after 3 hr, as shown in **Fig. 9a**. Moreover, GSK3 slows down the decay of E-cadherins and has no effect on expression of N-cadherins.

It was found that transcription rates of Axin increase after Wnt activation. The transcriptional feedbacks result in the periodic oscillations of cellular concentrations of Axin under continuous Wnt stimulations [66]. In order to capture the oscillatory behavior of Axin, an additional protein “inhibitor 2″ that inhibits the activation of Wnt receptors was added into the network [31]. The transcription rate of “inhibitor 2″ is positively regulated by the Wnt target genes (Table 3). Previous experimental studies also showed that as the concentration of Axin increases, the activities of DVL that mediates binding between Axin and Wnt receptor are inhibited [66]. Therefore, we further adjusted the model so that the binding between Axin and activated Wnt receptor is inhibited when the concentration of Axin increases,
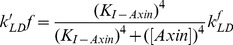
(3)



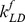
 is the rate constant defined in reaction 35 of Table 2, and 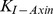 equals to 6.67 nM.

Consequently, our simulation results have not only demonstrated the oscillation of Axin, but also demonstrated the oscillations in destruction complex cycle (**Fig. 9b**). More interestingly, the simulation further shows a decayed oscillation in E- N-cadherin switch (**Fig. 9c**). The figure suggests that cells undergo switch from expression E-cadherin to N-cadherin during the first 4 hr. This switch will recover after long simulation time due to the decayed oscillation of other molecular components in the network. This phenomenon is difficult to obtain experimentally, because the simulations were generated under constant Wnt stimulation and all the other environmental factors were neglected. However, it provides general insights that the inhibition of E-cadherin after Wnt activation is oscillatory and decayed.

Overall, our computational model demonstrated the functional roles of transcriptional feedback in regulating the interplay between Wnt signaling and cadherin-mediated adhesion. Specific dynamic properties were archived, such as cadherin switch and Axin oscillation. The decayed oscillation in cadherin switch brings further insights to understand the mechanism of EMT.

## Discussions

In this article we constructed a network model to evaluate the dynamic interplay between cell adhesion and Wnt signaling pathway. The model integrates different functional modules of cadherin clustering, β-catenin degradation circle and transcriptional regulation. Our calibrated model reproduced the most recent experimental phenomena with semi-quantitative accuracy. We have computationally illustrated that Wnt signaling and cadherin-mediated cell adhesion crosstalk with each other through multiple pathways. Except the simple competition for β-catenins between these two systems, the slow releasing of ABCs from membrane associated destruction complexes caused by spatial organization of cell adhesion plays an additional role to inhibit Wnt signaling. Cell adhesion also affects Wnt signaling by regulating the phosphorylation rate of β-catenins. Adding transcriptional feedback loops into the model, we have further revealed a decayed oscillation during cadherin switch. Finally, our results demonstrated the importance of spatial information in regulating the dynamics of cellular signaling pathways.

In order to incorporate the spatial information, our model divided the signaling network into multiple layers or compartments according to the subcellular locations of different network components, including membrane surface, cytoplasm, Golgi and nucleus. The same type of molecules will be denoted by different states and possess of different properties if they translocate from one layer to another. For instance, only the active β-catenin in cell nucleus can trigger the target gene expression. Similarly, the destruction complexes change the properties of phosphorylation after they bind to the Wnt receptors. However, the protein-protein interactions within each layer or compartment are still assumed to take place in a well-mixed, homogeneous medium according to our simulation design. This assumption is limited by the fact that proteins tend to be spatially organized into specific patterns at subcellular locations. The cadherin clustering in adherens junctions is one of the best examples. In our cell adhesion module, we have introduced a network model to partially compensate the heterogeneity of cadherin clustering and its interaction with β-catenins. In this adhesion network model, clustering is simplified by a transition process. Junctions form if a larger portion of cadherin-catenin complexes transit from “*trans*” state to “*cis*” state. Although it was shown theoretically that receptor clustering is a phase transition from a diluted state to a condensed state, the details of many kinetic features, such as the cooperativity between cadherin’s *trans* and *cis* interactions, cannot be quantitatively taken into account by current model. More sophisticated spatial simulation techniques are expected to resolve this issue. The interactions, aggregations and organizations of proteins in specific cellular environments are well approached by particle-based simulation methods [54]. The incorporation of these methods into our model will be helpful to spatial-temporally elucidate the functional impacts of cadherin-based cell adhesion to Wnt signaling pathway.

Several aspects of the Wnt pathway are still poorly understood. Firstly, the molecular mechanism from Wnt receptor activation to the engagement of intracellular partners remains elusive. The “initiation-amplification” model suggests that there is cooperation between LPR6 receptor phosphorylation and Axin recruitment [67]. The “signalosome” model was further proposed based on the observation that Wnt induced aggregation of LRP6 receptors [68]. Both models gave consistent perspective that clustering of receptors upon Wnt activation amplifies the signal transduction. However, further validation of these models has to wait until high-resolution structures of Wnt proteins in complex with FZD and LPR6 are achieved. Secondly, the model of β-catenin nuclear transport has been a matter of debate [69]. It has been proposed that β-catenins are carried by APC when they translocate through the nucleic pore. Yet, considering the structural similarity between β-catenin and importin/exportin, it has also been suggested that the import and export of β-catenin are independently of any nuclear transport receptor. Thirdly, genetic assays have identified many of β-catenin’s nuclear binding partners. Some of these factors are involved in chromatin structure and RNA polymerase II regulation [70]. The diverse natures of these interactions make it difficult to elucidate a cohesive picture of β-catenin-mediated transcriptional regulation. In conclusion, detailed analysis of all these factors is beyond the scope of the present study. Future extension can be reached by developing multi-scale models with specific purposes. For instance, a coarse-grained model has been constructed for the functional state of nuclear pore complex and Brownian dynamic simulation was used to study the nucleo-cytoplasmic transport [71]. The rate constant derived from this model for β-catenin can be integrated into our model to evaluate the kinetic property of β-catenin nuclear transport.

It has been demonstrated that the concentrations of Wnt signaling proteins in different cell types is significantly different [34]. The functioning loops of the Wnt signaling and cadherin-mediated adhesion pathways may also differ between different organisms and cell lines. Furthermore, other signaling pathways such as notch [72] or EGF [73] can bring additional effects through the signaling crosstalk. Therefore, while focusing on the interplay between cadherin adhesive and Wnt signaling processes, the model we proposed in this work only serves as a generic and flexible framework. It can be modified to characterize specific experimental scenarios by changing the values of the model parameters, such as the reaction rates and initial concentrations in different cell types. The model can also be modified to incorporate additional phenomena, such as crosstalks with other cell signaling pathways. Finally, observations of Wnt signaling suggest that β-catenin levels and TCF-dependent transcription oscillate during the cell cycle [74]. The key components of the pathway including Axin, GSK3, and APC have been shown to associate with centrosomes and/or kinetochores during mitosis. Accumulating experimental evidences, together with the future development of computational modeling, should allow qualitative predictions to be made about the relationship between Wnt activity levels and the cell cycle.

## Conclusions

The Wnt signaling pathway is critical to induce the epithelial-mesenchymal transition, in which the cadherin-based cell adhesion is significantly repressed. Recent experimental studies revealed that Wnt signaling and cadherin-mediated cell adhesion interplay with each other through multiple pathways. The simulation results from our computational model gave supportive evidences that cell adhesion affects Wnt signaling in both negative and positive ways. Cadherins can inhibit Wnt signaling not only in a way as a stoichiometric binding partner of β-catenins that sequesters them from signaling, but also in a way through their clustering to impacts the rate at which β-catenins are involved in the destruction loop. In contrast, cadherin clustering also increases the phosphorylation rate of β-catenins and promotes its signaling in nucleus. Finally, adding transcriptional regulation into the model helps us to understand the mechanism of EMT.

## Supporting Information

Document S1 The full mathematical description of the model.
(DOCX)
