# Supplementary material for: Computational Modeling of the Interplay between Cadherin-Mediated Cell Adhesion and Wnt Signaling Pathway
Source: PLoS One. 2014 Jun 26;9(6):e100702. doi: 10.1371/journal.pone.0100702 (PMC4072676; doi:10.1371/journal.pone.0100702)
Supplement: Document S1 — The full mathematical description of the model. (DOCX) [file pone.0100702.s001.docx]

**Supporting Document S1**

***Full mathematical representation of the model***

1. **Cadherin-based cell adhesion**

*Synthesis of Cad/Cat Complex:*

$\begin{matrix} \frac{d([Cad/Cat]_{go\lg i})}{dt}=f([ABC/TCF]_{Cad})S_{Cad/Cat} \\ -k_{shuttle}[Cad/Cat]_{go\lg i}-k_{\deg}^{BC}[Cad/Cat]_{go\lg i} \end{matrix}$ [S1]

*Regulation of Cad/Cat Complex on Cell Membrane:*

$\begin{matrix} \frac{d([Cad/Cat]_{free})}{dt}= \\ k_{shuttle}[Cad/Cat]_{go\lg i}-k_{endo}^{free}[Cad/Cat]_{free} \\ +(k_{BC,f}^{free}[B-Cat][Cad]_{free}-k_{BC,b}^{free}[Cad/Cat]_{free}) \\ -(k_{trans}^{f}[Cad/Cat]_{free}-k_{trans}^{b}[Cad/Cat]_{free}) \end{matrix}$ [S2]

$\begin{matrix} \frac{d([Cad/Cat]_{trans})}{dt}= \\ (k_{BC,f}^{trans}[B-Cat][Cad]_{trans}-k_{BC,b}^{trans}[Cad/Cat]_{trans}) \\ +(k_{trans}^{f}[Cad/Cat]_{free}-k_{trans}^{b}[Cad/Cat]_{trans}) \\ -k_{endo}^{trans}[Cad/Cat]_{trans}-(k_{cis}^{f}[Cad/Cat]_{trans}-k_{cis}^{b}[Cad/Cat]_{cis}) \end{matrix}$ [S3]

$\begin{matrix} \frac{d([Cad/Cat]_{cis})}{dt}=(k_{BC,f}^{cis}[B-Cat][Cad]_{cis}-k_{BC,b}^{cis}[Cad/Cat]_{cis}) \\ +(k_{cis}^{f}[Cad/Cat]_{trans}-k_{cis}^{b}[Cad/Cat]_{cis})-k_{endo}^{cis}[Cad/Cat]_{cis} \end{matrix}$ [S4]

$\frac{d([Cad]_{cis})}{dt}=k_{BC,b}^{cis}[Cad/Cat]_{cis}-k_{BC,f}^{cis}[B-Cat][Cad]_{cis}-k_{endo}^{cis}[Cad]_{cis}$ [S5]

$\frac{d([Cad]_{trans})}{dt}=k_{BC,b}^{trans}[Cad/Cat]_{trans}-k_{BC,f}^{trans}[B-Cat][Cad]_{trans}-k_{endo}^{trans}[Cad]_{trans}$ [S6]

$\frac{d([Cad]_{free})}{dt}=k_{BC,b}^{cis}[Cad/Cat]_{free}-k_{BC,f}^{cis}[B-Cat][Cad]_{free}-k_{endo}^{free}[Cad]_{free}$ [S7]

1. **Wnt stimulation and β-catenin degradation**

*Retention of B-cat caused by Apc and Axin.*

$\begin{matrix} \frac{d([Axin])}{dt}=f([ABC/TCF]_{axin})S_{axin}-d_{axin}[Axin] \\ -(k_{AP/AX,f}[Axin][APC]-k_{AP/AX,b}[Axin/APC]) \\ -(k_{AX/B,f}[Axin][B-Cat]-k_{AX/B,b}[Axin/B-Cat]) \end{matrix}$ [S8]

$\begin{matrix} \frac{d([APC])}{dt}=S_{apc}-d_{apc}[APC]-(k_{AP/AX,f}[Axin][APC] \\ -k_{AP/AX,b}[Axin/APC])-(k_{AP/B,f}[APC][B-Cat] \\ -k_{AP/B,b}[APC/B-Cat]) \end{matrix}$ [S9]

*Formation of destruction complex*

$\begin{matrix} \frac{d([Axin/APC])}{dt}=(k_{AP/AX,f}[Axin][APC]- \\ k_{AP/AX,b}[Axin/APC])-(k_{D,f}[Axin/APC][GSK3]-k_{D,b}[D]) \end{matrix}$ [S10]

$\begin{matrix} \frac{d([D])}{dt}=(k_{D,f}[Axin/APC][GSK3]-k_{D,b}[D])- \\ (k_{BD,f}[D][B-Cat]-k_{BD,b}[BD])+k_{re\mathrm{cov}}[ID]-k_{LD}^{f}[D][WC] \end{matrix}$ [S11]

*Destruction complex cycle*

$\begin{matrix} \frac{d([BD])}{dt}=(k_{BD,f}[D][B-Cat]-k_{BD,b}[BD])- \\ (k_{p,WNT-OFF}^{CK1}[BD]-k_{dp,WNT-OFF}^{CK1}[CK1-pBD])-k_{LD}^{f}[BD][WC] \end{matrix}$ [S12]

$\begin{matrix} \frac{d([CK1-pBD])}{dt}=(k_{p,WNT-OFF}^{CK1}[BD]-k_{dp,WNT-OFF}^{CK1}[CK1-pBD]) \\ -(k_{p,WNT-OFF}^{GSK3}[CK1-pBD]-k_{dp,WNT-OFF}^{GSK3}[GSK3-pBD]) \\ -(k_{release,WNT-OFF}^{ABC}[CK1-pBD]-k_{ass,WNT-OFF}^{ABC}[ABC][D]) \\ -k_{LD}^{f}[CK1-pBD][WC] \end{matrix}$ [S13]

$\begin{matrix} \frac{d([GSK3-pBD])}{dt}=(k_{p,WNT-OFF}^{GSK3}[CK1-pBD]-k_{dp,WNT-OFF}^{GSK3}[GSK3-pBD]) \\ -k_{transform}[GSK3-pBD]-k_{LD}^{f}[BD][GSK3-pBD] \end{matrix}$ [S14]

$\begin{matrix} \frac{d([GSK3-pB/ID])}{dt}=k_{transform}[GSK3-pBD] \\ +k_{transform}[GSK3-WBD]-k_{\deg radation}[GSK3-pB/ID] \end{matrix}$ [S15]

$\frac{d([ID])}{dt}=k_{\deg radation}[GSK3-pB/ID]-k_{re\mathrm{cov}}[ID]$ [S16]

*Wnt stimulation*

$\begin{matrix} \frac{d([WC])}{dt}=S_{WC}+k_{transform}[GSK3-WBD]-k_{\deg}^{WC}[WC] \\ -k_{LD}^{f}[WC][D]-k_{LD}^{f}[WC][BD]-k_{LD}^{f}[WC][CK1-pBD]-k_{LD}^{f}[WC][GSK3-pBD] \end{matrix}$ [S17]

$\frac{d([WD])}{dt}=k_{LD}^{f}[WC][D]-(k_{BD,f}[WD][B]-k_{BD,b}[WBD])$ [S18]

$\begin{matrix} \frac{d([WBD])}{dt}=k_{LD}^{f}[WC][BD]+(k_{BD,f}[WD][B]-k_{BD,b}[WBD]) \\ -(k_{p,WNT-ON}^{CK1}[WBD]-k_{dp,WNT-ON}^{CK1}[CK1-WBD]) \end{matrix}$ [S19]

$\begin{matrix} \frac{d([CK1-WBD])}{dt}=k_{LD}^{f}[WC][CK1-pBD] \\ +(k_{p,WNT-ON}^{CK1}[WBD]-k_{dp,WNT-ON}^{CK1}[CK1-WBD]) \\ -(k_{p,WNT-ON}^{GSK3}[CK1-WBD]-k_{dp,WNT-ON}^{GSK3}[GSK3-WBD]) \\ -(k_{release,WNT-ON}^{ABC}[CK1-WBD]-k_{ass,WNT-ON}^{ABC}[ABC][WD]) \end{matrix}$ [S20]

$\begin{matrix} \frac{d([GSK3-WBD])}{dt}=k_{LD}^{f}[WC][GSK3-pBD] \\ +(k_{p,WNT-ON}^{GSK3}[CK1-WBD]-k_{dp,WNT-ON}^{GSK3}[GSK3-WBD]) \\ -k_{transform}[GSK3-WBD] \end{matrix}$ [S21]

*Wnt signal transduction*

$\begin{matrix} \frac{d([ABC])}{dt}=(k_{release,WNT-ON}^{ABC}[CK1-WBD]-k_{ass,WNT-ON}^{ABC}[ABC][WD]) \\ +(k_{release,WNT-OFF}^{ABC}[CK1-pBD]-k_{ass,WNT-OFF}^{ABC}[ABC][D]) \\ -(k_{p}^{Y142}[ABC]-k_{dp}^{Y142}[ABC/Bcl9]) \end{matrix}$ [S22]

$\begin{matrix} \frac{d([ABC/Bcl9])}{dt}=(k_{p}^{Y142}[ABC]-k_{dp}^{Y142}[ABC/Bcl9]) \\ -(k_{NT}^{f}[ABC/Bcl9]-k_{NT}^{b}[ABC/Bcl9(N)]) \end{matrix}$ [S23]

$\begin{matrix} \frac{d([ABC/Bcl9(N)])}{dt}=(k_{NT}^{f}[ABC/Bcl9]-k_{NT}^{b}[ABC/Bcl9(N)]) \\ -(k_{BT}^{f}[ABC/Bcl9(N)]-k_{BT}^{b}[ABC/TCF]) \end{matrix}$ [S24]

$\frac{d([ABC/TCF])}{dt}=k_{BT}^{f}[ABC/Bcl9(N)]-k_{BT}^{b}[ABC/TCF]$ [S25]
